# Prevalence of Overweight and Obesity in Urban Elementary School Children in Northeastern Romania: Its Relationship with Socioeconomic Status and Associated Dietary and Lifestyle Factors

**DOI:** 10.1155/2013/537451

**Published:** 2013-07-14

**Authors:** Veronica Mocanu

**Affiliations:** Department of Pathophysiology, Grigore T. Popa University of Medicine and Pharmacy, 700115 Iasi, Romania

## Abstract

The purpose of this paper is to estimate the prevalence of obesity and to identify its potential determinants to optimize the methods of prevention to combat further increases in childhood overweight. The study was carried out on 3444 school children of 6–10 years of age attending 30 schools in northeast Romania. Schools were classified by geographical location and socioeconomic status (SES). Overweight and obesity status were determined using IOTF BMI cut-off points. Prevalence of overweight (including obesity) was found to be 24.6% among boys and 22.6% among girls, whereas the prevalence of obesity was 7.8% in boys and 6.3% in girls. High SES (OR: 1.46; 95% CI: 1.10–1.93) and eating French fries and chips (OR: 1.81; 95% CI: 1.24–2.67) were associated with increased risk of overweight. In high- and medium-SES children, overweight was positively associated with the consumption of French fries and chips (2.93, 95% CI: 1.54–5.60 and 1.82, 95% CI: 1.04–3.21). In low-SES children, overweight was associated with low fruit consumption (0.21, 95% CI: 0.05–1.00) and sedentary behavior (3.37, 95% CI: 1.13–10.05). Therefore, the social and environmental determinants should be considered when constructing and implementing preventive measures regarding overweight and obesity.

## 1. Introduction

Childhood obesity is increasingly becoming a public health concern in developing countries. Significant nutritional, demographic, epidemiological, and socioeconomic transitions occurred during the last two decades in Eastern European Countries [1, 2]. After the collapse of communism, Romania experienced significant global influences of obesogenic environment which relates globalization, urbanization and westernization to enhancing access to nontraditional foods. Changes in patterns of behaviors, such as physical activity, sedentary behavior, and dietary practices, especially associated with urban-industrial life styles, have been found to contribute to the rise in childhood obesity [2–7].

Increasing burden of obesity, the metabolic syndrome, type 2 diabetes mellitus, and cardiovascular diseases in developing countries has created an urgent need for population-based strategies to tackle childhood obesity. Clearly, these efforts require understanding of the modifiable factors that influence obesity and the metabolic syndrome in developing countries [6].

Romania is currently experiencing a nutrition transition with increased intake of foods rich in carbohydrates, saturated fat, and cholesterol [2, 3, 7] and an increase in sedentary lifestyles [3, 7]. Despite these trends, there is no data on diet- and activity-related behaviors associated with overweight and obesity among Romanian children.

In order to develop behavioral interventions targeted at improving dietary habits and help prevent obesity in elementary school-aged children, information about the dietary habits and lifestyle is required [8–10]. Thus, the objectives of this study were (1) to investigate the current prevalence of overweight and obesity in a large sample of children living in northeastern Romania and (2) to identify the modifiable factors associated with an increased risk of obesity in children aged 6–10 years.

## 2. Methods

### 2.1. Participants

A population-based cross-sectional study was conducted among elementary school children within the Healthy Traditions programme that is part of the EPODE International Network (EIN). Schools in the Iasi and Neamt counties, Northeast Romania, were purposively sampled from small (<10,000 inhabitants) to medium sized (10,000–50,000 inhabitants) and large sized (50,000–300,000 inhabitants) cities. The multistage stratified cluster random sampling method was applied to obtain selected schools. There were 30 schools in total within four cities (Iasi, Roman, Targu Frumos, and Podu Iloaie). The listed schools were stratified according to the geographic area and socioeconomic status (SES). SES information was obtained from the Iasi and Neamt School Inspectorates. Based on this information, the schools were stratified by SES (average monthly household income) to low, medium, and high (<10,000, 10,001–25,000, and >25,001 Romanian lei, resp.) [1 Euro = 4.4 Romanian lei]. Ten schools, respecting geographical location and socioeconomic status, were selected to be included in the lifestyle survey. 

Head teachers at selected schools were first sent an invitation letter and an information leaflet and then contacted for a meeting. During the meeting, details were provided about the aims of the study, the invitation and measurement procedures, the degree of school involvement required, and potential benefits of participation. An information pack was provided at the same time, containing a copy of the ethical approval, examples of all study materials, and information leaflets for all teachers. 

Each participating child was required to obtain written, informed consent from his or her legal guardian before they could take part in the measurement procedures. Ethical approval for this study was obtained from the Grigore T. Popa University of Medicine and Pharmacy ethics committee.

### 2.2. Procedures

#### 2.2.1. Data Collection

Sampled schools were visited for screening the students for anthropometric measurements on prearranged dates during the months of October and November between 2008 and 2012. All research staff members completed a training program conducted by the Principal Investigator that familiarized them with the specific tools and methods used. 

#### 2.2.2. Anthropometric Measurements

Height was measured to the nearest 0.1 cm with a portable stadiometer (KaWe, Germany), and weight was measured in light clothing to the nearest 0.1 kg on a digital scale (Healthy Line, SHL-9015B, CE). All anthropometric data were collected by trained staff and supervised by the school nurse. Final height and weight values were obtained as means of the two or three measurements. BMI was calculated as weight (kg) divided by squared height (m^2^). Overweight and obesity were defined using the international body mass index cut-off points established for children [11]. These cut-off points are based on health related adult definitions of overweight (≥25 kg/m^2^) and obesity (≥30 kg/m^2^) but are adjusted to specific age and sex categories for children [11]. Waist circumference was measured to the nearest 0.1 cm at the midpoint between the lower costal border and the top of the iliac crest with the measurement taken at the end of a normal expiration.

#### 2.2.3. Lifestyle Variables

Data pertaining to lifestyle habits were collected via a self-report questionnaire administered in ten selected schools. At the school level, a response rate of 87% was achieved. Due to limited resources, we chose to self-administer the questionnaire as this makes it possible to collect simultaneously a large quantity of information from many subjects in a short period of time, costs less to administer than personal interview, and requires fewer trained personnel. The questionnaire was self-administered during school time under the supervision of the teacher. In order to minimize the possibility of bias, all supervisors had received two hours of instruction about the questionnaire and were standardized in answering any of the students' questions if explanations were needed. We used an adapted version of the CATCH Kids Club After-School Student Short Questionnaire [12], a valid and reliable survey instrument [13]. A pilot study with a sample of Romanian children 6–10 years old to assess the cross-cultural suitability of the questionnaire demonstrated its appropriateness and the full understanding of questions content by the children. No modifications were required; thus construct validity was assumed. Questions were designed to assess behaviors related to physical activity (sports participation), sedentary habits (time spent watching television and computer usage), and dietary practices (consumption of fried food, confectionary, vegetables, fruits, and fruit juice). Potential answers for the sports question “Yesterday, did you exercise at least 20 minutes” were “0,” “1,” “2,” or “3 or more” times. Answers for “During the week/weekend, how many hours per day do you usually spend watching TV/playing video games/or using the computer to surf the Internet” were “0,” “1,” “2,” or “3 or more” hours.

For each of these variables, responses were dichotomized: “less than two” and “more than two” except physical activity: “less than one” and “more than one.”

Answers for “Yesterday, did you eat” French fries or chips, fruit and vegetable, fruit juice, or confectionery were “0,” “1,” “2,” or “3 or more” times. 

#### 2.2.4. Statistical Analysis

The results were analyzed statistically using the Statistical Package for the Social Sciences (SPSS, version 15.0). Descriptive statistics were carried out on the data generated. Continuous data are reported as means ± standard deviations, while categorical data are presented as percentages. The Pearson's chi-squared test was used to determine eventual differences between prevalence in each group. The impact of social and environmental determinants on the prevalence of overweight/obesity was analyzed by using odds ratios (ORs), established by binary logistic regression adjusted for the age included in the regression models.

## 3. Results

### 3.1. Recruitment

The study included 3687 school children in the age group of 6–10 years; 1845 were boys and 1842 were girls. Children with biologically implausible (or extreme) values were excluded from the analysis (6.6%). In all, 3444 children (1712 boys and 1732 girls) were included in this study.

Table 1 shows the physical characteristics of the study sample grouped according to age and sex. The height was significantly (*P* < 0.001) higher in obese/overweight as compared the normal weight ones. There were no significant sex differences when all age groups were combined.

### 3.2. Prevalence of Overweight and Obesity

Table 2 shows the prevalence of overweight (not including obesity) and obesity in each age and sex group. Overall, 16.8% of boys and 16.3% of girls were overweight with a further 7.8% of boys and 6.4% of girls classified as obese. The prevalence of overweight and obesity was greater in boys than girls but it was not significantly different (*P* = 0.11). The prevalence of overweight and obesity among children aged 6–10 years was significantly (*P* < 0.05) higher in 7- and 9-year-old children than in 10 year-old ones. 

### 3.3. Relationships between Prevalence of Overweight and Obesity and SES

**Figure 1:**
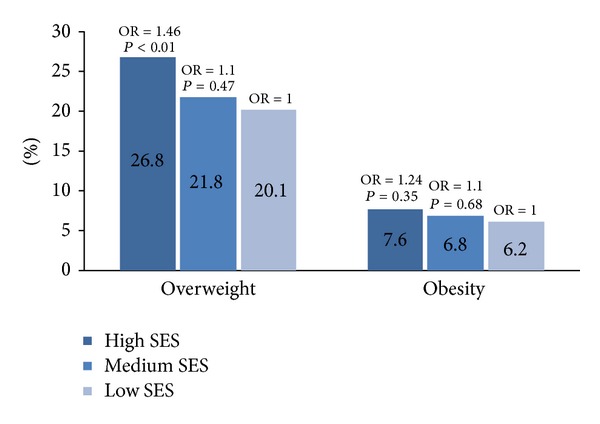
Overall prevalence (%) of overweight (obesity included) and obesity and relationships between socioeconomic status (SES) and overweight or obesity among Romanian children (*n* = 1015). Logistic regression was used to assess the association between overweight/obesity versus normal-weight and obesity versus normal-weight as dependent variable and SES groups as independent variables. OR = odds ratio.

The overall prevalence of overweight and obesity and its relationship with the socioeconomic status are presented in Figure 1. The prevalence of overweight among children (both sexes) was 19.2% in high-SES group, 14.9% in medium-SES group, and 13.8% in low-SES group; the prevalence of obesity was 7.7% in high-SES group, 6.8% in medium-SES group and 6.2% in low-SES group. Logistic regression was used to assess the association between overweight/obesity versus normal-weight and obesity versus normal-weight as dependent variable and SES groups as independent variables. No significant differences between girls and boys for any SES groups.

### 3.4. Relationships between Overweight/Obesity and Risk Determinants

Table 3 shows the crude and adjusted odds ratio (OR) of the risk determinants for overweight/obesity. On binary logistic regression analysis of all the group, the risk of overweight/obesity was found significantly higher (1.46, 95% CI: 1.11–1.93, *P* < 0.01) in high-SES children compared with low-SES children. The consumption of French fries and chips was found to be significantly associated with overweight (1.80, 95% CI: 1.22–2.65, *P* < 0.01). The consumption of vegetables, fruits, fruit juice, and confections showed no associations with the odds of overweight/obesity in our sample. 

The association between overweight and physical activity was not statistically significant in our study. Furthermore, no associations were found significant between overweight and time spent on watching TV or using computer, either in boys or girls.

### 3.5. Relationships between Overweight/Obesity and Lifestyle Risk Factors according to SES

Table 4 displays the potential determinants of overweight/obesity differentiated by SES groups. In high SES group, overweight was positively associated with the consumption of French fries and chips (2.93, 95% CI: 1.54–5.60, *P* < 0.001) and confectionery (1.63, 95% CI: 1.00–2.66, *P* < 0.05). In medium-SES group, overweight was positively associated only with the consumption of French fries and chips (1.82, 95% CI: 1.04–3.21, *P* < 0.01). In low-SES group, overweight was associated with decreased consumption of fruits (0.21, 95% CI: 0.05–1.00, *P* < 0.05), fruit juice (0.34, 95% CI: 0.67–1.70, *P* < 0.01), and increased daily time spent watching television and using the computer (3.37, 95% CI: 1.13–10.05, *P* < 0.05).

## 4. Discussion

To our knowledge, this is the first study from Romania to identify socioeconomic and lifestyle factors associated with overweight in 6- to 10-year-old boys and girls. Also, this is the first study from the northeastern part of Romania to assess the prevalence of overweight and obesity using an appropriate and commonly accepted definition of childhood overweight and obesity (International Obesity Task Force) [11].

Among children of primary school age, data collected from European countries (based on the IOTF definitions and measured data) showed a large variation with a prevalence of overweigth (including obesity) from 11 to 37% in boys and from 15 to 35% in girls, while the prevalence of obesity varied from 2 to 14% in boys and from 3 to 12% in girls [14]. In Central and Eastern Europe, the countries with the highest prevalence of overweight (including obesity) were Slovenia (overweight 25%, obesity 8%), Bulgaria (overweight 20%, obesity 6.6%), and Lithuania (overweight 16%, obesity 5.1%) [14]. In Romania, the high prevalence of overweight and obesity in children living in urban areas was previously reported in other regions of the country: in western Romania (overweight 25%, obesity 7.2%) by Chirita Emandi et al. [15] and in central Romania (overweight 21%, obesity 8%) by Valean et al. [16]. The present study showed that the prevalence of overweight was high among children living in urban areas in northeastern Romania, with 24.6% in boys and 22.7% in girls. Meanwhile, obesity was observed in 7.8% of boys and 6.4% of girls, which is alarmingly close to the highest obesity rate observed for Central and Eastern Europe (8%). 

The results of this study place Romania among the countries with a high prevalence of obesity, making research and implementation of obesity prevention and reduction programs a priority. The high prevalence of overweight and obesity in Romanian elementary school children could be explained by a number of factors including socioeconomic status as well as the availability/preferences for food and leisure-time physical activities [17]. 

It has been previously reported that BMI is influenced by different socioeconomic status (SES) backgrounds [18, 19]. Our analysis showed that childhood obesity is significantly related to SES. On logistic regression analysis, the risk of obesity and overweight was significantly higher in high-SES children compared with low-SES children. The higher the SES the more the risk of obesity in Russia and China [18], while in USA and western Europe [20, 21], the lower the SES, the higher the risk of obesity. Possible explanations for the different SES-overweight and obesity relationship in the countries undergoing an economic transition such as Romania are the lifestyle and nutritional determinants such as decreased physical activities, sedentary lifestyle, altered eating patterns, and increased fat content of the diet [22]. The widespread ownership of televisions, computers and cars, and the supermarket system of food retail all facilitate obesogenic behavior. In Romania, such ownership is quite recent, and more differentiated by class than in the United States and Western Europe [1, 23]. Any obesity prevention interventions may well influence social categories differently [20].

Of all the environmental variables assessed in all group, only consumption of French fries and chips followed expected trends [24, 25]. The consumption of vegetables, fruits, fruit juice, and confectionery showed no associations with the odds of overweight/obesity in our sample. Physical activity, and sedentary lifestyle showed no association with overweight when all the group was analyzed.

Our study examined the differences in dietary habits, physical activity, and sedentary lifestyle between children of high and low SES. When comparing SES groups, the consumption of French fries and chips was positively associated with overweight and obesity in high- and medium-SES children from the studied urban areas. In low-SES children, overweight was negatively associated with the consumption of fruit and fruit juice and positively associated with the time spent on watching television and using the computer. The sedentary behavior affected especially the low-SES boys, who have a high percentage of obesity. The disparity in risk determinates for obesity between high- and low-SES children could be due to cultural and environmental differences such as the lifestyle habits or opportunities to exercise due to societal constraints [19]. The Romanian culture celebrates a larger body ideal, and traditional diets are rich in carbohydrates (bread, potatoes) and animal products. The high rate of obesity found in Romanian boys may reflect a desire for “bigness,” muscularity, strength or, masculinity [26]. Further research should be undertaken to establish whether this acceptance of obesity may also reflect social class conditions, class distinction, nutritional education, occupation or, other social factors.

The current study provided initial insights into elementary school children patterns and weight status in Romania. A particular strength of the study is the fact that the prevalence of overweight and obesity was explored using an international standard (International Obesity Task Force). Our findings need to be viewed in the light of several limitations. One limitation is the cross-sectional design, which introduces uncertainties regarding the sequence of cause and effect of the observed associations. This study focused on children living in urban areas from two counties in northeastern Romania and may not be nationally representative. On the other hand, we consider that the possibility of a selection bias in relation to lifestyle habits is low. The potential for selection bias (only one third of the schools enter the lifestyle survey) was reduced through our population-based design and high response rate. Self-report methods are known to be subject to considerable recall bias and have limited validity and reliability among children. However, we minimized such bias by similar instructions for the teachers as well as ensuring children of the confidentiality of their responses. This also reduces desirability bias among respondents. The investigators acknowledge the need for tighter measurement control procedures to be included in a second study. Nonetheless, the findings from this study provide information about the health of children aged 6–10 years living in Romania and indicate that children require urgent interventions that target multiple aspects of diet, physical activity, and sedentary behaviors.

## 5. Conclusions

In conclusion, the data revealed that the proportion of elementary school children who were overweight (obesity included) and obese reached alarming rates (24% and 7%, resp.). We found that the risk of overweight was significantly higher in high-SES children compared with low-SES children and risk determinants differ remarkably with different socioeconomic status. Eating French fries and chips in high-SES children and low fruit consumption and sedentary behavior in low-SES children were independently associated with the risk of overweight. Further understanding of socioeconomic influences on overweight and obesity in our country is vital for planning and implementing effective prevention initiatives that will in turn foster a healthy environment for our children.

## Figures and Tables

**Table 1:** Participant characteristics (*n* = 3444).

	Height (cm)	Weight (kg)	Waist circumference (cm)	Body mass index (kg/m^2^)
6 yrs				
M (*N* = 278)	122.3 (5.8)	24.0 (5.0)	58.2 (6.0)	16.0 (2.3)
F (*N* = 277)	121.7 (5.6)	23.7 (4.8)	57.1 (5.8)	15.9 (2.3)
Total (*N* = 555)	**122.0 (5.7)**	**23.8 (4.6)**	**57.6 (6.0)**	**15.9 (2.3)**
7 yrs				
M (*N* = 441)	127.0 (7.1)	27.2 (5.7)	59.0 (6.6)	16.8 (2.8)
F (*N* = 461)	126.1 (6.8)	26.2 (5.6)	58.1 (6.6)	16.4 (2.7)
Total (*N* = 902)	**126.6 (7.0)**	**26.7 (5.6)**	**58.6 (6.5)**	**16.6 (2.7)**
8 yrs				
M (*N* = 403)	132.3 (7.0)	29.9 (6.9)	61.2 (7.5)	17.0 (3.0)
F (*N* = 394)	131.2 (6.6)	28.5 (6.2)	59.3 (7.0)	16.4 (2.8)
Total (*N* = 797)	**131.6 (6.8)**	**29.2 (6.6)**	**60.3 (7.2)**	**16.7 (2.9)**
9 yrs				
M (*N* = 333)	137.3 (7.2)	34.0 (8.5)	64.2 (8.8)	17.9 (2.9)
F (*N* = 324)	136.6 (6.7)	32.9 (7.8)	62.8 (8.9)	17.5 (3.2)
Total (*N* = 657)	**137.0 (7.0)**	**33.5 (8.2)**	**63.5 (8.3)**	**17.7 (3.3)**
10 yrs				
M (*N* = 257)	143.3 (7.1)	37.1 (8.5)	65.3 (8.0)	17.9 (3.2)
F (*N* = 276)	142.2 (7.9)	35.4 (8.3)	63.4 (8.3)	17.4 (3.0)
Total (*N* = 533)	**142.7 (7.5)**	**36.2 (8.4)**	**64.3 (8.2)**	**17.6 (3.1)**

Data expressed as mean ± SD.

**Table 2:** Prevalence of overweight^a^ and obesity based on IOTF criteria [11] by age and sex (*n* = 3444).

	Males (%)		Females (%)		All (%)	
	Overweight	Obesity	Overweight	Obesity	Overweight	Obesity
6 yrs (*n* = 555)	13.2	5.3	16.7	7.1	14.9	6.2
7 yrs (*n* = 902)	14.6	10.0	16.8	8.1	15.8	9.1
8 yrs (*n* = 797)	17.8	7.4	14.0	6.3	15.2	6.5
9 yrs (*n* = 657)	19.1	9.4	19.1	6.5	16.0	6.9
10 yrs (*n* = 533)	18.6	4.7	14.8	3.2	16.6	3.9
All (*n* = 3444)	16.8	7.8	16.3	6.4	16.6	7.1

^a^Not including obesity.

Pearson's chi-squared test was used to compare categorical variables. No significant differences between girls and boys for any age. Age trend for overweight but not for obesity.

**Table 3:** Associations between overweight and socioeconomic status, dietary behaviors, physical activity, and sedentary lifestyle (*n* = 1015)^a^.

	Prevalence of overweight (obesity included)^b^, *n* = 244		OR (95%)	
	%	*P* value^b^	Crude	Adjusted^c^
Socioeconomic status (SES)				
Low SES	20.1	0.000	1	1
Medium SES	21.8		1.10 (0.83–1.46)	1.10 (0.83–1.47)
High SES	26.8		1.46 (1.10–1.93)**	1.46 (1.11–1.93)**
French fries and chips intake				
<2 times/day	22.1	0.000	1	1
≥2 times/day	34.0		1.81 (1.24–2.67)**	1.80 (1.22–2.65)**
Vegetable intake				
≥2 times/day	23.6	0.826	1	1
<2 times/day	24.1		1.035 (0.76–1.40)	1.035 (0.76–1.40)
Fruit intake				
≥2 times/day	23.6	0.259	1	1
<2 times/day	21.6		0.84 (0.61–1.14)	0.83 (0.60–1.14)
Fruit juice				
≥2 times/day	26.8	0.590	1	1
<2 times/day	24.7		0.86 (0.58–1.28)	0.86 (0.57–1.28)
Confectionery intake				
<2 times/day	23.6	0.841	1	1
≥2 times/day	23.4		0.98 (0.71–1.37)	0.99 (0.72–1.39)
Exercise (20 min/day)		0.898		
≥1 time/day	23.9		1	1
<1 time/day	23.5		0.97 (0.71–1.34)	0.99 (0.72–1.37)
Watching TV and playing PC				
<2 hours/day	23.4	0.960	1	1
≥2 hours/day	23.5		0.99 (0.72–1.36)	1.01 (0.73–1.38)

^a^Binary logistic regression with overweight/obesity versus normal weight as dependent variable and lifestyle factors as independent variables. The models are controlled for age of the child.

^b^According to the age and specific cut-off points for BMI as published by the IOTF [11].

^c^Adjusted: the model is adjusted for sex and age of the child.

**Significantly different from reference group (*P* < 0.01).

**Table 4:** Associations between overweight and dietary behaviors, physical activity, and sedentary lifestyle according to SES (*n* = 1015)^a^.

	Socioeconomic status (SES)		
	High SES (*N* = 416)	Medium SES (*N* = 417)	Low SES (*N* = 182)
Sex			
Females	1	1	1
Males	1.04 (0.82–1.32)	1.16 (0.91–1.47)	1.32 (0.79–2.21)
Fat snack intake			
<2 times/day	1	1	1
≥2 times/day	2.93 (1.54–5.60)***	1.82 (1.04–3.21)**	1.65 (0.42–6.43)
Vegetable intake			
≥2 times/day	1	1	1
<2 times/day	1.06 (0.69–1.62)	1.19 (0.72–1.95)	0.82 (0.23–2.97)
Fruit intake			
≥2 times/day	1	1	1
<2 times/day	0.90 (0.58–1.38)	0.78 (0.47–1.27)	0.21 (0.05–1.00)*
Fruit juice			
≥2 times/day	1	1	1
<2 times/day	0.94 (0.57–1.52)	1.24 (0.49–3.12)	0.19 (0.05–0.73)**
Confectionery intake			
<2 times/day	1	1	1
≥2 times/day	1.63 (1.00–2.66)*	0.97 (0.58–1.60)	0.34 (0.67–1.70)
Exercise (20 min/day)			
≥1 time/day	1	1	1
<1 time/day	1.04 (0.55–1.82)	1.42 (0.82–2.49)	1.71 (0.38–7.7)
Watching TV and playing PC			
<2 hours/day	1	1	1
≥2 hours /day	1.14 (0.67–1.91)	1.04 (0.65–1.66)	3.37 (1.13–10.1)*

^a^Binary logistic regression with overweight/obesity versus normal weight as dependent variables and lifestyle factors as independent variables by SES. The models are controlled for age of the child. *Significantly different from reference group (*P* < 0.05). **Significantly different from reference group (*P* < 0.01). ***Significantly different from reference group (*P* < 0.001).
